# Effect of an edge at cup rim on contact stress during micro-separation in ceramic-on-ceramic hip joints

**DOI:** 10.1016/j.triboint.2017.01.012

**Published:** 2017-09

**Authors:** Feng Liu, John Fisher

**Affiliations:** a School of Mechanical and Power Engineering, North University of China, PR China; b Institute of Medical and Biological Engineering School of Mechanical Engineering, University of Leeds,UK

**Keywords:** Ceramic-on-ceramic, Hip joint replacement, Edge contact, Micro-separation, Finite element

## Abstract

Alumina ceramic total hip joint bearings have shown superior wear properties. The joint bearing may undergo adverse conditions such as micro-separation causing head contact on the cup rim. As a transition, an edge is formed between the cup bearing and the rim. The aim of this study was to predict the effect of the edge on contact stresses in order to better understand the mechanisms of wear. A finite element contact model was developed under the conditions of the head displacements 0.5–2 mm and vertical loads 0.5–3 kN. The edge contact produced the most severe stresses capable of causing elevated wear and damage to ceramic bearings. The study shows that the bearing design should be considered in association with clinical conditions to eliminate severe stress.

## Highlights

• Head-cup rim contact due to micro-separation was studied for ceramic total hip joints.
• An edge formed between the cup bearing surface and the cup rim was analysed.
• Finite element model was developed to predict contact stress distribution and intensity.
• A non-smooth edge led to substantially increased stress concentration and high contact stress.
• The resulting high contact stresses can lead to ceramic bearing wear and damage.

## 1 Introduction

Hip joint replacements are an effective surgical procedure to treat patients with joint diseases such as osteoarthritis [1]. However, excessive wear particles resulting from the joint bearing contact may cause inflammatory responses and consequently implant failure, and remains a limiting factor for hip prostheses to achieve long-term performance [2], [3]. In terms of the bearing materials, ceramic-on-ceramic (CoC) bearing couples exhibit superior wear properties when compared to metal-on-metal (MoM) or metal-on-polyethylene (MoP) combinations [4], [5], [6], [7], [8], [9], [10]. In addition, ceramic wear debris has shown to be less biologically active [11], [12], [13]. Therefore, an increasing number of current hip joints use CoC bearings and the development of CoC bearings have become of a great interest.

Contact mechanics plays an important role in determining wear mechanism of hip implants. An ideal contact of artificial hip joints require the bearing components being properly positioned to assure contact area produced at the cup bearing surface not intersecting cup rim [13], [14]. However, adverse conditions such as micro-separation may cause head-cup rim contact and consequently high stress and substantially elevated wear [15], [16], [17]. Micro-separation has been associated with varied clinical situations including head offset deficiency, medialized cup, stem subsidence and soft tissue laxity [13]. These conditions can cause the femoral head to be positioned laterally relative to the cup, and can be compounded as a result of joint motion [18]. Fluoroscopic studies showed dynamic separation of the cup and head [19]. Nevelos et al. [15] proposed a mechanism that may occur in a gait cycle. For example, during swing phase, when the load is low, the head is lateralized due to laxity of the joint, femoral head offset deficiency or medialized cups, making contact at the cup rim. When the load increases at heel strike, it causes edge loading and the head sliding back into the centre of rotation of the cup. Laboratory simulator tests have shown that micro-separation produces stripe-like wear on the head and at the cup rim for CoC hip joint bearings [20], [21], [22], [23], [24]. A displacement of 0.5 mm of the head produced wear and wear particle distributions which replicates clinical wear patterns [20], [21], [22], [23]. Further simulator studies also showed that surgical factors such as cup inclination angles and the magnitudes of micro-separation displacement can play a part in the elevated wear [22], [23].

Computational studies using finite element (FE) methods have been used to help understand wear mechanism of head-cup rim contact for total hip joint replacements [16], [25], [26], [27], [28]. Mak et al. [16] predicted elevated contact stress as a result of micro-separation for CoC bearings but they were not able to carry out a convergence study pointing out the limitation in obtaining accurate stress values due to the sensitive nature of the model for edge contact. Scifert et al. [29] developed models to study influencing factors for MoM hip dislocation. Elkin et al. [25], [30] used a similar model to study the effect of subluxation and impingement on stress concentration for MoM bearings and fracture mechanics for CoC hips. Previously, the present authors predicted the contact stress for MoM bearings under micro-separation rim contact conditions, in which a substantially refined FE mesh was used to improve the convergence of the model [27]. Sanders and Brannon [31] proposed a method based on Hertzian contact theory particularly for predicting contact stress at the rounded section of the cup rim. In the clinical design of ceramic cup bearing, a transition from the spherical bearing surface to the cup rim is inevitable and the process of manufacturing produces a non-smooth edge at the cup rim with a discontinuous slope at the edge. The contact due to this singularity may cause more severe stress concentration than that of the rounded section of the cup rim. An accurate prediction of contact stress at the edge can provide more insights on wear generation for CoC bearings under adverse conditions such as micro-separation rim contact.

For CoC hip joint, the typical materials used include alumina ceramic and zirconia-toughened alumina [23]. The materials are much stiffer with higher Young's modulus of 360–380 GPa compared to 230 GPa for the metal [31] which means that a further refined FE mesh is needed to capture the smaller deformation and highly concentrated stress for CoC bearings. The non-smooth edge poses particular difficulties for stress predictions. The focus of present study is to provide an accurate stress prediction based on a theoretical edge design with geometric singularity at cup rim in order to better understand the mechanisms of wear and damage.

## 2 Material and methods

**Fig. 1:**
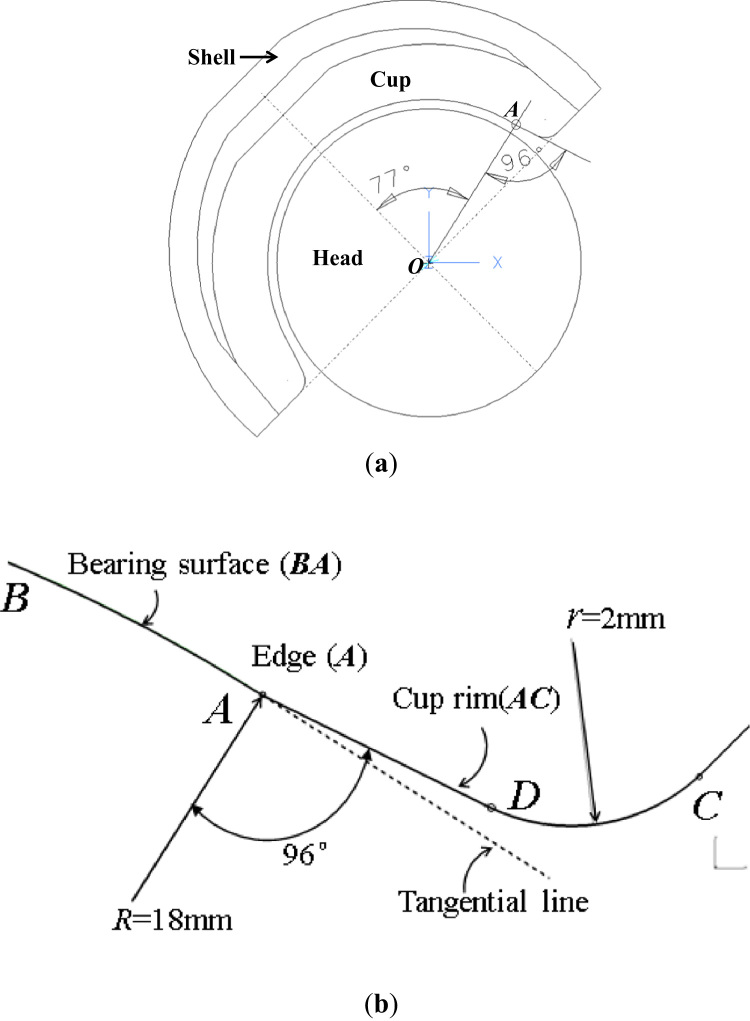
Cross-sectional view of ceramic-on-ceramic total hip joint replacement, consisting of an acetabular cup with an inclination angle of 45°, a femoral head and a titanium backing shell. An edge is formed at the cup rim (point *A*) and located at 77° relative to the pole of the cup (a). The details of the cup rim with the edge, including a straight line segment (*AD*) and a circular segment (*DC*) (b).

**Fig. 2:**
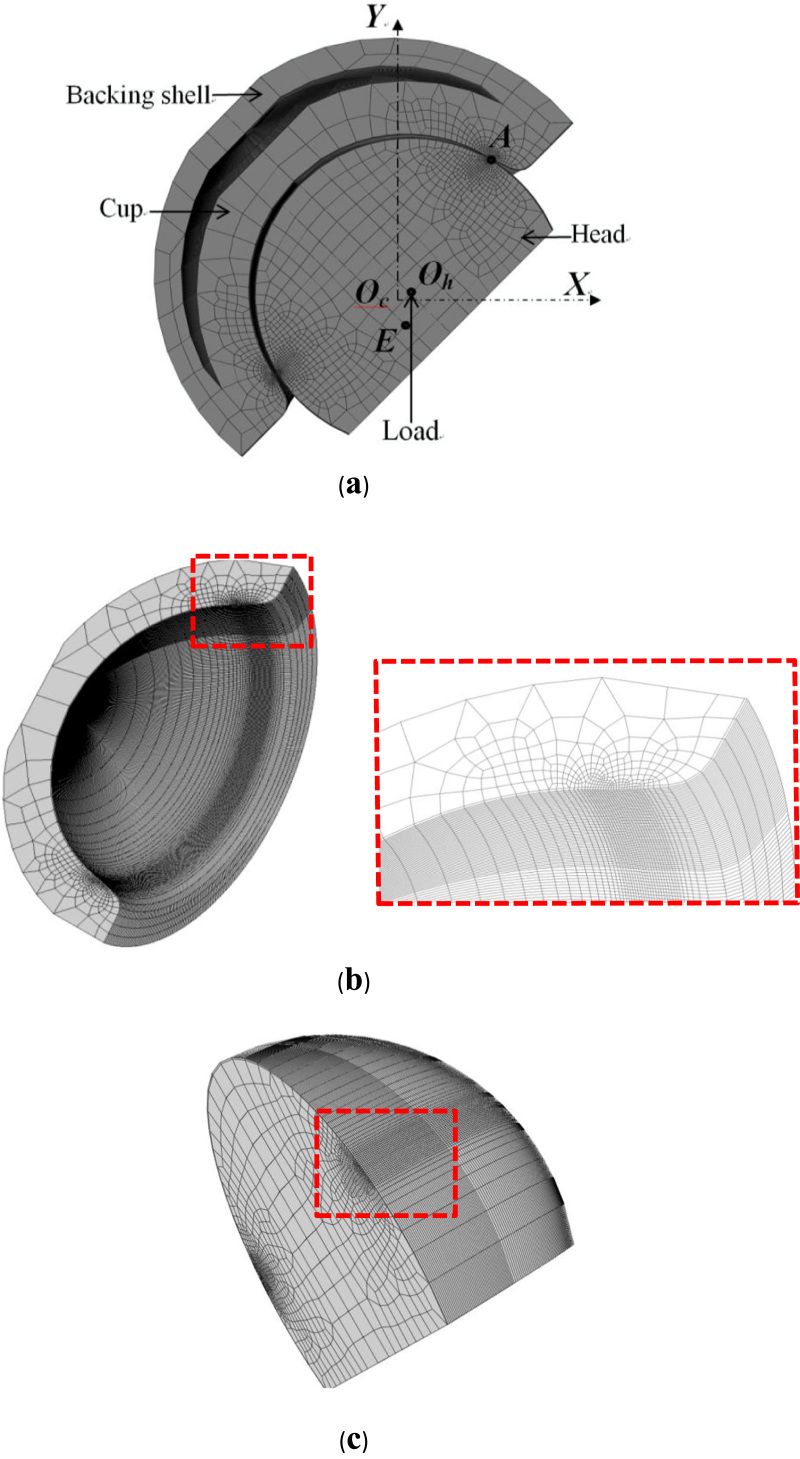
Finite element contact model (half) for rim contact at the edge (point *A*), with a vertically applied load at the head centre (*O*_h_) as a result of the lateral displacement of the head (0.5 mm, the distance in the *X* direction between *O*_h_ and *O*_c_, the centre of ration of the cup) (a). A detailed view of the cup (b) and head (c) with the refined meshes for the rim contact.

Total hip joints are typically of ball-in-socket configuration consisting of a hemispherical acetabular cup articulating against a spherical femoral head with a low clearance between the bearing surfaces of the cup and head. A 36-mm diameter ceramic-on-ceramic (alumina-alumina) hip joint bearing with a titanium backing shell was considered as shown in Fig. 1a. The cup is positioned with an inclination angle of 45° and press-fitted into the backing shell through a taper connection. The anteversion of the cup was simplified as zero for the present study. The bearing geometry was based on the engineering drawing of a clinical design (Pinnacle, DePuy Synthes, UK). The details of the cup rim are illustrated in Fig. 1b. For the cup rim, an edge (located at point *A*) is formed between the spherical bearing surface (*BA*) and an adjacent conical section (*AD*) which is at 96° relative to the radial line *OA* (Fig. 1b), and the half coverage angle of the cup bearing is 77°. Head contact with the cup rim was modelled as a result of micro-separation which is characterized as the lateral translational displacement of the head centre relative to the centre of rotation of the cup leading to contact at the edge (point *A*) as shown in Fig. 2a.

A three-dimensional FE model was created with the first order hexahedral elements (C3D8) for the rim contact formulation in ABAQUS (Version 6.11-1; Dassault Systems Simulia Corporation, Providence, RI) (Fig. 2a). A quasi-static analysis was carried out to simulate rim contact at a discretised time instant with a vertical load being applied at the head centre. The outer surface of backing shell was fully fixed to represent an ideal implant-bone fixation. The interface at the taper connection between the ceramic liner and the backing shell was considered as fully bonded. The nodal points at the head centre (*O*_h_) and an adjacent point *E* were chosen to be applied with the boundary condition of displacements as being constrained in the horizontal direction (*OX*) to prevent rigid body motion. In order to reduce computational time, a half FE model was used (Fig. 2a) by making use of the symmetry of full model but the displacements of nodes on the symmetrical plane (*OXY*) were constrained in the direction (*OZ*) perpendicular to the symmetry plane. The major geometric and mechanical property parameters used for the model are summarized in Table 1.

**Table 1.** Dimensions and mechanical properties of ceramic-on-ceramic bearing (For the shell, the radius is for the outer surface).

	Radius (mm)	Young's modulus (GPa)	Poisson's ratio
Cup	18.0	380	0.26
Head	17.965	380	0.26
Shell	27	110	0.3

The FE mesh was generated in NX I-deas 6.1 (Siemens PLM Software, TX). Alumina ceramics with Young's modulus 380 GPa and Poisson's ratio 0.26, and the titanium shell with Young's modulus 110 GPa and Poisson's ratio 0.3 were considered [16]. The effect of friction on contact stress was found to be negligible for static contact analysis. The mesh sensitivity and convergence check was conducted with a locally refined mesh especially designed for the contact region on both the cup and head (Fig. 2a-c). The contact element size of 0.5 mm was chosen and repeatedly halved to a minimum of 0.0008 mm. The vertical load range of 0.5–3 kN was considered. The convergence study was carried out with the load of 0.5 kN and the lateral displacement of 0.5 mm. In order to carry out parametric studies, the element size of 0.0625 mm was chosen to predict the trend for comparison for a given micro-separation displacement range of 0.5–2 mm with the load in the range of 0.5–3 kN. The corresponding number of elements was approximately 330,000 for the half model, and the model with a single load was run for 3 h of computing time on a computer of 2.8 GHz, 12 Gb RAM.

## 3 Results

**Fig. 3:**
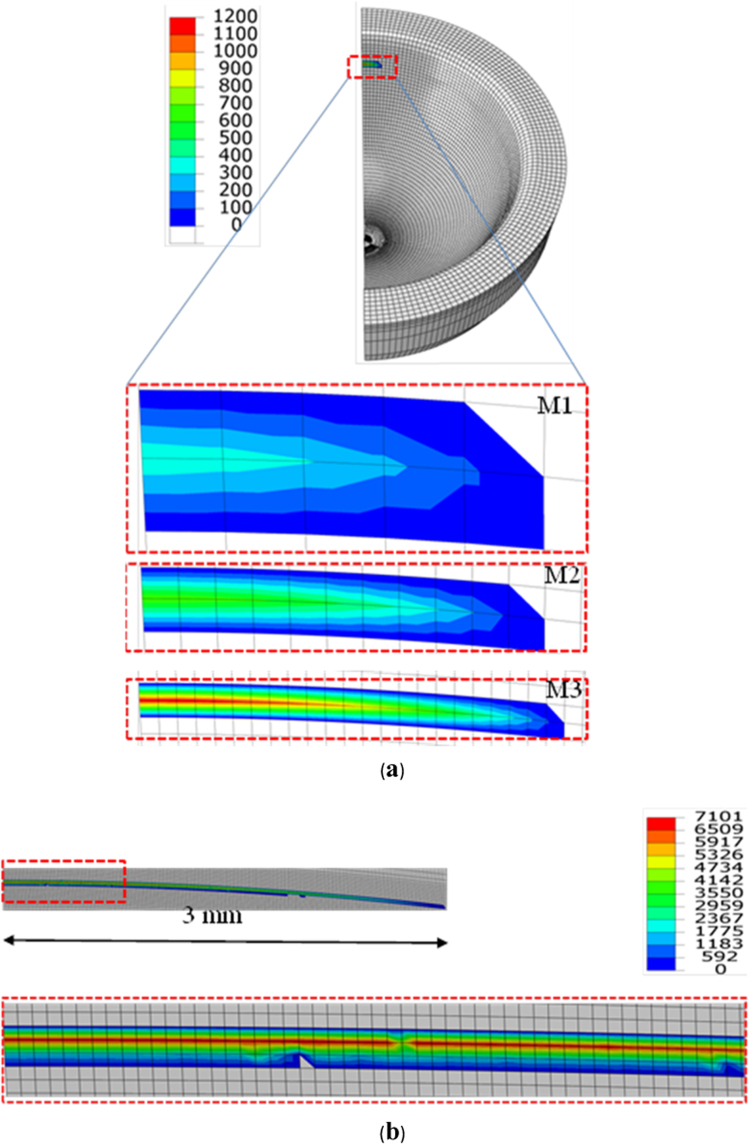
Comparison of computationally predicted contact pressures (MPa) distributed along the edge on the cup rim for three representative mesh densities with the element sizes of 0.5, 0.25 and 0.0125 mm, as a result of head lateral displacement of 0.5 mm under a load of 0.5 kN (a). The contact pressure distribution along the edge (approximately 3 mm in length) for the mesh with the element size 0.0156 mm, and a close view of the distribution for the first quarter length as highlighted (b).

Contact pressures were predicted to distribute in a stripe area along the edge on the cup rim (half model) with the half length of contact at 2.5–2.87 mm using the FE model of varied meshes with the contact element sizes reduced from 0.5 to 0.125 mm (Fig. 3a). A highly concentrated line contact was predicted along the edge as shown by the contact pressure distribution obtained from a further refined mesh with the element size of 0.0156 mm (Fig. 3b). The dimension of contact area along the edge was slightly increased (3 mm) while the dimension in the perpendicular direction was considerably reduced (0.0468 mm). The stress concentration resulted in much higher contact pressures (7 GPa) as predicted using the finer meshes (Fig. 3b).

**Fig. 4:**
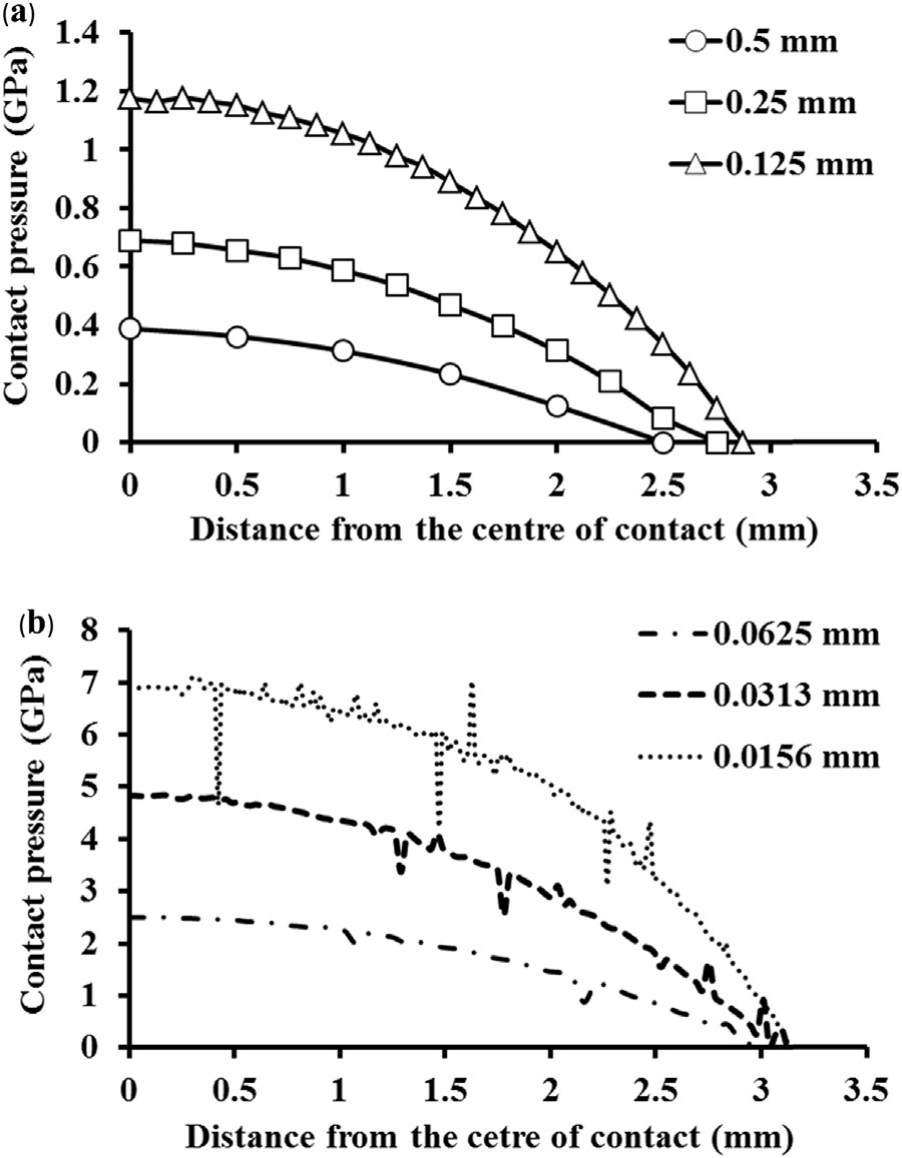
Comparisons of computationally predicted contact pressures along the edge as a function of the distance from the centre of contact (point *A*) on the cup rim, for different mesh densities, with the element sizes of 0.5, 0.25, and 0.125 mm (a), and 0.0625, 0.0313 and 0.0156 mm (b), respectively, for the head displacement of 0.5 mm and load of 0.5 kN.

The predicted contact pressure values along the edge were plotted as a function of the distance from the centre of contact and compared for varied mesh densities, with the element sizes reduced from 0.5 to 0.125 mm (Fig. 4a) and from 0.0625 to 0.0156 mm (Fig. 4b). The corresponding maximum contact pressures were found to increase from 0.4 to 7 GPa. However, the finer meshes with the element sizes of 0.0625, 0.0313 and 0.0156 mm showed numerical oscillation in contact pressure (Fig. 4b). This was due to the slightly mismatching mesh at the interface between the edge and the head to achieve point-to-point contact in obtaining smooth contact pressure predictions [27].

The FE mesh was further improved with the element size sequentially reduced to 0.0008 mm. The corresponding maximum contact pressures and tensile stresses are listed in Table 2. The peak contact pressures and tensile stresses were as high as 31.78 and 18.51 GPa, respectively. The differences in contact pressure between two consecutive mesh densities were reduced to approximately 20% while for tensile stress they stayed approximately at 50%.

**Table 2.** Finite element mesh density check based on the lateral displacement of the head 0.5 mm under the vertical load of 0.5 kN.

Element size (mm)	Maximum contact pressure (GPa) and difference percentage		Maximum tensile stress (GPa) and difference percentage	
0.25	0.69		0.11	
0.125	1.18	42%	0.19	42%
0.0625	2.50	53%	0.36	47%
0.03125	4.85	48%	0.67	46%
0.015625	7.10	32%	1.36	51%
0.0078	12.34	43%	2.94	54%
0.0033	21.30	42%	6.67	56%
0.0016	25.75	17%	9.36	29%
0.0008	31.78	19%	18.51	49%

**Fig. 5:**
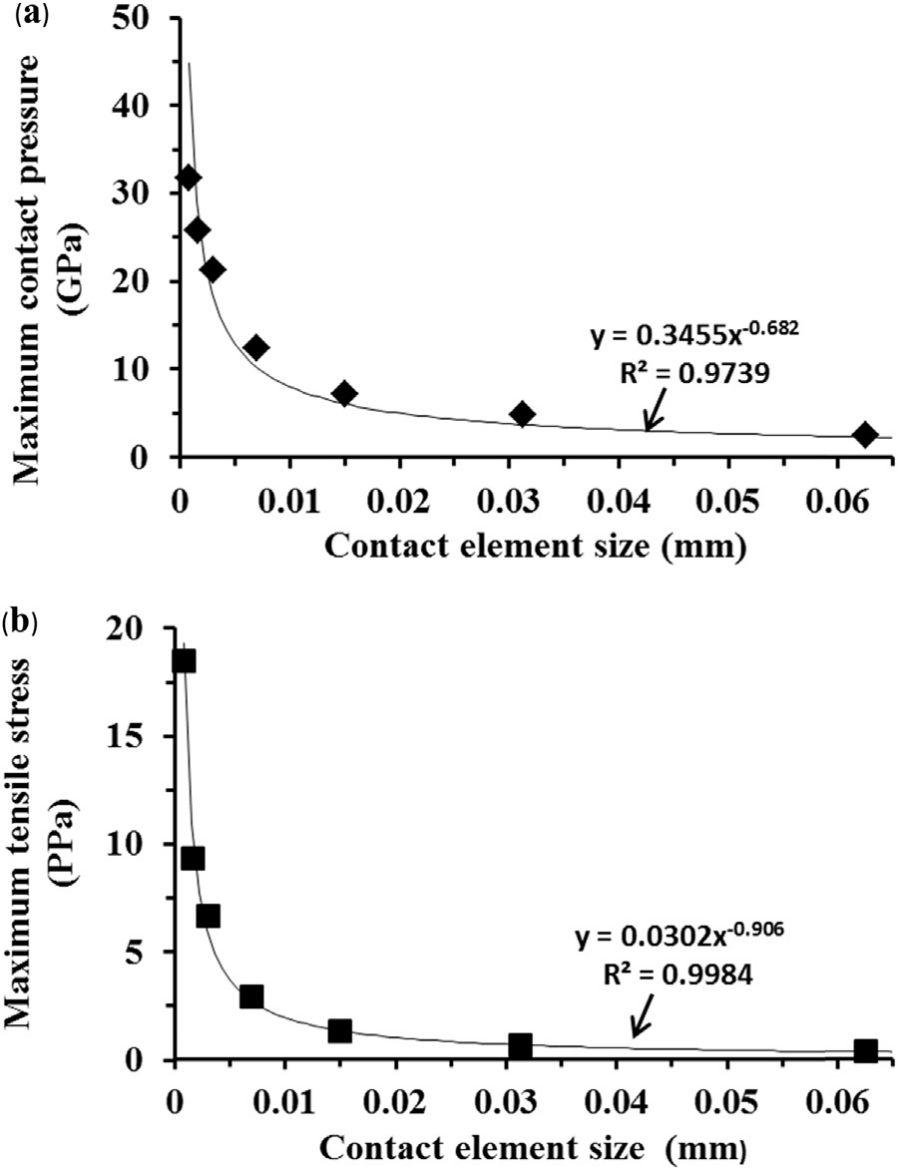
Computationally predicted maximum contact pressures (a) and maximum tensile stresses (b) as a function of the element size of the contact regions for the head lateral displacement of 0.5 mm and load of 0.5 kN. The curve-fitted functions are also superimposed, respectively.

The maximum contact pressure and maximum tensile stress for varied mesh densities were found to be a function of the element size, as curve-fitted by *y*=0.3455*x*^−0.682^ and *y*=0.0302*x*^−0.906^, respectively (Fig. 5a and b). The functions indicate that both the maximum contact pressure and maximum tensile stress tended to increase further with the mesh density increased. This means an infinite stress for the edge contact and was due to the no smooth edge geometry and its discontinuous gradient. According to theoretical contact mechanics [32], a high stress concentration would be expected if contact surfaces are discontinuous in the slope of profiles. The present FE model provides a consistent prediction with that of theoretical contact mechanics.

**Fig. 6:**
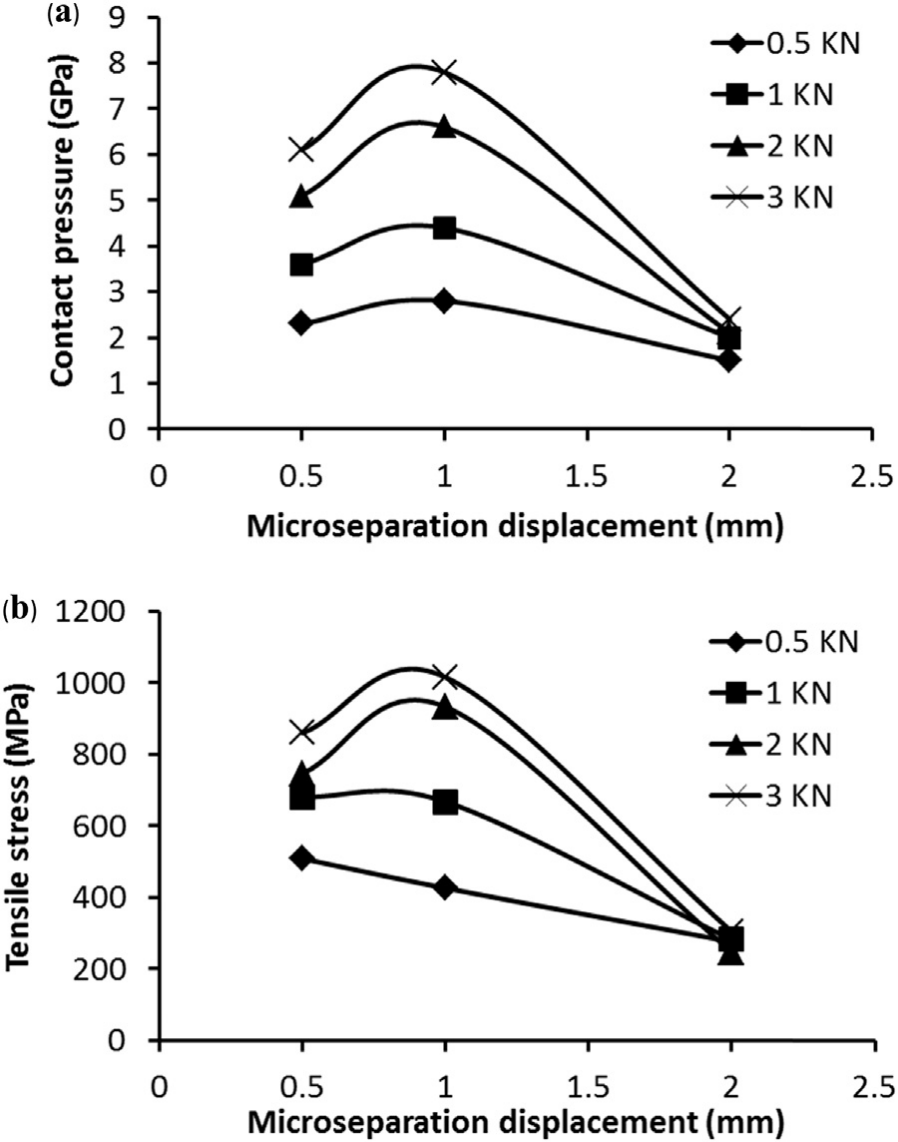
Computationally predicted maximum contact pressures (a) and maximum tensile stresses (b) as a function of the head displacements of 0.5–2 mm for the mesh with the contact element size 0.0625 mm under loads 0.5–3 kN, respectively.

**Fig. 7:**
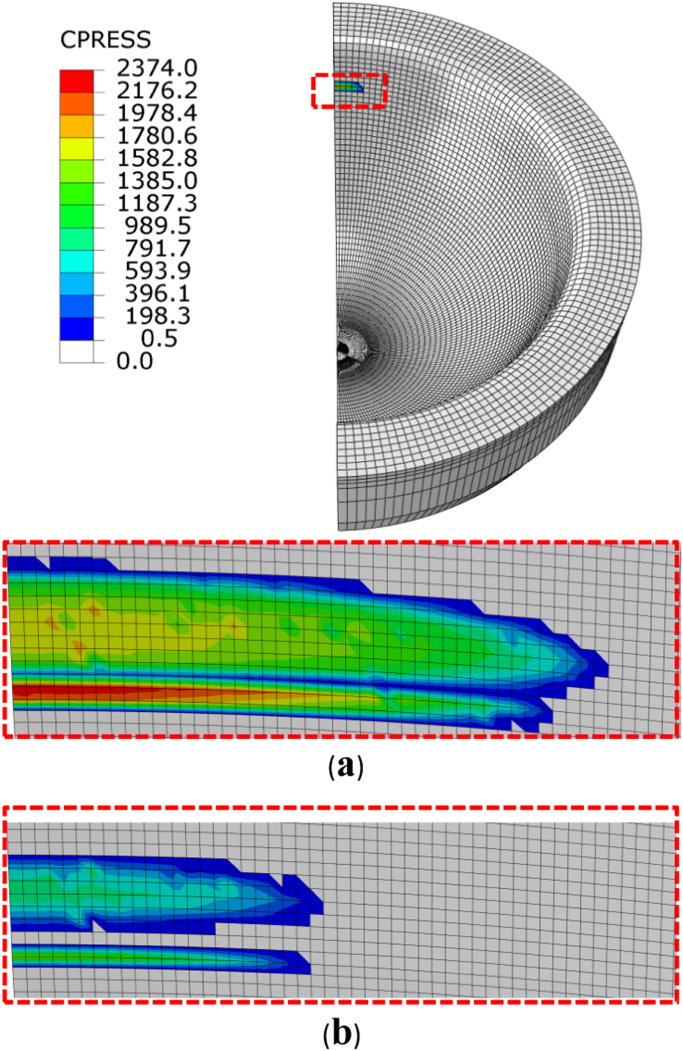
Comparison of computationally predicted contact pressures distributed along the edge on the cup rim for the head lateral displacement of 2.0 mm under the loads of 0.5 kN (a) and 3 kN (b), respectively. For the mesh with the element size of 0.0625 mm, the lengths of contact area are approximately 2.7 and 1.4 mm, and the maximum contact pressures are 2374 and 1519 MPa for the loads of 0.5 and 3 kN, respectively).

For comparison, the mesh density with the element size of 0.0625 mm for the edge was chosen to investigate the effect of micro-separation displacements on contact stresses. The maximum contact pressures and maximum tensile stresses were predicted for micro-separation with the head lateral displacement in the range of 0.5–2 mm under loads increased from 0.5 to 3 kN (Fig. 6a and b). The displacement of 1 mm of the head was found to produce the largest contact pressures and tensile stresses. The increase in displacement to 2 mm led to larger contact area, reduced stress concentration and lower contact pressures (Fig. 7) compared with those of the lower displacement of 0.5 mm (Fig. 3b).

## 4 Discussion

A non-smooth transition in geometry of the cup bearing surface produces an edge at the cup rim (Fig. 1b). Theoretically, contact surfaces having non-continuous gradient in profiles will produce high stress concentration [32], with the discontinuous slope at the edge of contact or within the contact interface. This paper is the first FE study in which a substantially finer FE mesh has been developed to capture infinitly high stresses as associated with a non-smooth edge at the cup rim for ceramic hip joint replacement. This study shows that micro-separation with the lateral displacement of 0.5 mm of the head led to contact between the head and the edge at the cup rim (Fig. 3). The contact pressures were found to be highly concentrated at the edge along a line as illustrated by the FE model using substantially refined mesh (Fig. 3b). The convergence study of the FE model showed that the magnitudes of contact pressures distributed along the edge were increasing with finer meshes (Fig. 4a and b). In particular, the maximum contact pressure and maximum tensile stress were found to be a function of the element size, the magnitudes of pressure (stress) being approximately proportional to 1/*x*, where *x* is the element size (according to the powers of −0.682 and −0.906, as curve-fitted for contact pressures and tensile stresses, respectively, in Fig. 5a and b). The indication of infinitely high contact pressure and tensile stress highlights the significant effect of the edge on contact stresses.

This edge loading condition was found to be analogous to that of a blunt wedge pressed into contact with an semi-infinite solid with elastic contact. In linear elasticity, contact pressure approaches infinity at the apex of the edge due to the discontinuity in the slope of contact geometry [32]. The present FE prediction was found to be consistent with that of the wedge model in terms of the infinitely high contact stresses at the edge (Fig. 4, Fig. 5). For alumina ceramics with high Young's modulus and hardness, the edge contact poses difficulties for FE model to obtain accurate prediction of the contact tresses. This study shows that to capture this highly concentrated stress, the FE mesh density needs to be substantially increased as indicated by the increasing maximum contact pressures and tensile stresses (Table 2). In reality, materials will yield plastically at a finite stress. The prediction in the strength of stress singularities would provide useful information about the intensity of stress concentration which helps provide better understanding of wear mechanisms.

Micro-separation causing rim contact for hip joints is a dynamic process. The occurance and severity of rim contact is dependent on several variables including cup inclination angles, translational malposition, soft tissue tension, cup design and bearing materials. Hip joint simulators have been used to investigate the effects of the variables on wear resulting from rim contact. But the study of contact mechanics in relation to the dynamic behaviour of micro-separation hip joints requires computational simulation. Previously, a virtual dynamic model of a hip joint simulator was developed to predict the severity of edge loading for CoC bearings [33]. The model showed that the load on the cup rim increased from 300 N to 3 kN when the magnitude of micro-separation displacement increased from 0.1 to 3.5 mm and the cup inclination angle increased from 35 to 55° as for clinically relavent conditions. The effects of micro-separation displacements and corresponding loads on contact stresses therefore should be considered in the wide range of displacements, loads and cup angles.

For the increased micro-separation displacements, the contact pressure was found to reach the maximum with the lateral displacement of the head at 1 mm, compared with that of the displacement at 2 mm (Fig. 6a). Similar trend was also found with the maximum tensile stress (Fig. 6b). The displacement of the head at 1 mm produced the most severe contact (Fig. 6) as a result of contact area being fully concentrated on the edge similar to that of the displacement of 0.5 mm. The contact pressure reduced for the displacement at 2 mm was due to the centre of contact being shifted away from the edge resulting in increased contact area (Fig. 7). For micro-separation with larger displacements (>1 mm), the edge contact modelled corresponding to the lower displacements (0.5–1 mm) should be considered as part of the whole dynamic micro-separation as the head slides back making full contact at the edge after heel strike.

For alumina ceramic considered in this study with the flexural strength approximately of 500 MPa [16], the predicted maximum tensile stresses (Fig. 6b) resulting from the micro-separation and edge contact were found to largely exceed the limit. This indicates intragranular fracture and pullout of the grains. Contact-induced damage of alumina ceramics have also been reported [34] for which the high contact pressure led to onset of inelastic deformation and microcracking in the subsurface region of high compression. Repeat contacts resulted in severe mechanical fatigue and the detachment of grains from the surface [34]. Therefore, both contact pressure and tensile stress are important to consider the effect of the edge designs on wear generation as well as damage to the bearing. In the present study，with the lateral displacement of the head in the range of 0.5–2 mm, contact at the non-smooth edge was predicted with infinitly high stress values, and the high contact stresses can cause damage to the rim [34]. This indicates that the original geometry of the edge would be altered due to damage as well as wear. The contact model should be further developed to incorperate the modified rim geometry. The modification of rim geometry should be linked with wear and damage resulting from rim contact. Presently, the incorperation of a wear and damage model is a challenge which needs a further development for the compuational model. The inelastic deformation of ceramic liner resulting from highly concentrated stresses was not incorperated in the present study, which is partly due to lack of detailed plastic deformation data of the ceramic material, and more due to wear and damage that can occur and lead to considerable modification of rim geometry. A contact mechanics simulation based on rim geometry measured after wear and damage will be considered in the further study.

The titanium alloy backing shell has been considered to deform with elastic deformation only as the load was transmitted through the taper connection which can be distributed over a relatively large contact area without causing plastic deformation. However, the press-fit interaction at the taper connection was not considered in the present study which can be a topic in a further study.

There are some limitations in the present study. As mentioned above, micro-separation rim contact is dynamic process which may need a dynamic model to incorperate the variables as many as possible such as friction, dynamic loads and varied kinematics. Some other variables including cup inclination and anteversion angles and bearing sizes and varied combinations of the variables were not analysed. A larger separation up to 4 mm is also clinically relevant which requires a further investigation. This study has been based on a theoretical design of the bearing before manufacturing. An advanced contact model should be developed to incorperate wear of the bearing due to rim contact which is necessary to provide a validation against experimental wear measurements.

## 5 Conclusions

An edge formed as a result of geometry transition between the cup bearing surface and the cup rim can lead to stress concentration at the edge under micro-separation conditions of hip joint bearings. The edge contact produced substantially high contact stresses beyond the flexural strength and compression strength of alumina ceramics used in the current CoC bearings. The prediction of contact stress at the edge requires the FE model with substantially refined mesh. For the current cup rim design, the micro-separation displacements at lower level 0.5–1 mm were found to produce highly concentrated contact between the edge and the head resulting in the most severe contact stresses. The design as well as manufacturing of the cup rim should be considered in association with adverse clinical conditions such as dynamic separation to eliminate severe stress and improve wear for CoC bearings.
